# Rapid Pituitary Apoplexy Regression: What Is the Time Course of Clot Resolution?

**DOI:** 10.1155/2015/268974

**Published:** 2015-03-15

**Authors:** Devon L. Jackson, Jamie J. Van Gompel

**Affiliations:** ^1^College of Medicine, Howard University, Washington, DC 20059, USA; ^2^Department of Neurological Surgery, Mayo Clinic, Rochester, MN 55905, USA

## Abstract

A 29-year-old male patient with a functioning pituitary macroadenoma is discussed. The pituitary mass was detected by MRI after the patient presented with sudden onset of headache, suggestive of an apoplectic event. The headache resolved with analgesic medications. Within a follow-up period of one week, the pituitary mass had spontaneously regressed to nearly half its original size without any therapy. The patient never reported any visual complaints and displayed no signs of hypopituitarism. Elevated prolactin levels were present. Seven weeks after the initial event, the pituitary mass showed continued regression on MRI. Prolactin levels remained elevated. This case provides a unique look at the rapid spontaneous regression of mass effect that may occur following apoplexy of a pituitary adenoma.

## 1. Introduction

Apoplexy into a prolactinoma is not rare and is more likely to occur in macroprolactinomas. In those that hemorrhage occurs, the majority of patients are women with an average age at diagnosis of 31, ranging from 25 to 37. Men have an average age at diagnosis of 41, ranging from 32 to 54 [1]. Pituitary tumor apoplexy is a rare event with common presenting signs and symptoms. Depending on the severity of the symptoms and signs, conservative or surgical treatment is implemented. In rare cases, treatment may be unnecessary as the pituitary tumor can spontaneously regress. Although the hemorrhage may resolve and involute on its own, the time course is unknown. Here we present a case of rapidly regressive pituitary apoplexy and potential time course for clot resolution.

## 2. Case Presentation

**Figure 1:**
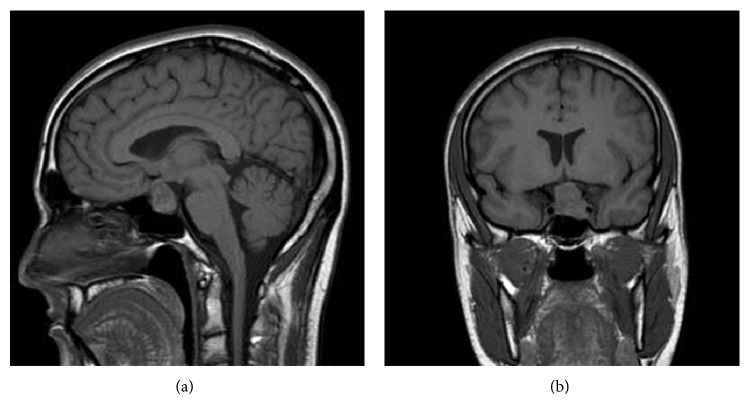
MRI, without contrast, revealed an enlarged sellar lesion on (a) sagittal T1-weighted and (b) coronal T1-weighted images. The optic chiasm cannot be clearly visualized in the coronal T1-weighted image.

A 29-year-old man presented with history of fatigue and sudden onset of headache on the left side of his head. The fatigue had been present for roughly six months. The headache occurred more recently. The patient reported that the headache felt like a migraine and was severe enough that he went to the emergency room. An MRA was done which was negative. CT of the head revealed a questionable enlarged pituitary gland. An MRI without contrast was then done which showed a mass, 2.5 cm in maximal diameter (Figure 1), arising from the sella turcica on the left side with some suprasellar extension with optic chiasmal elevation. At the time, the patient received Dilaudid and his headache resolved. Visual field testing had not been done yet, but no visual complaints were noted. The patient also had no signs of acromegaly, hypothyroidism, or diabetes insipidus. A lumbar puncture was performed in the ER which was negative.

**Figure 2:**
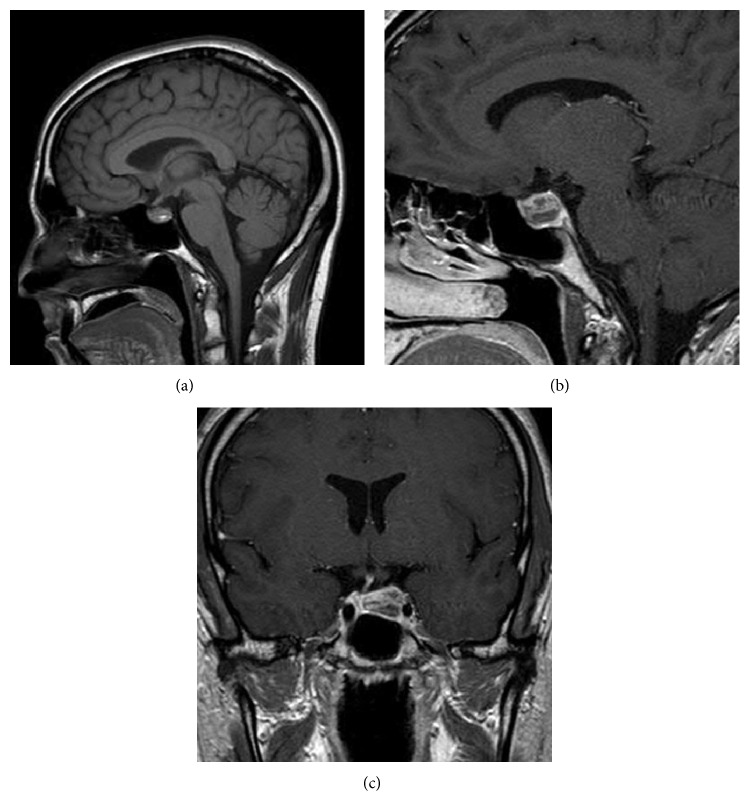
One week after the MRI in Figure 1, MRI revealed a shrinking sellar lesion on (a) sagittal precontrast T1-weighted, (b) sagittal postcontrast T1-weighted, and (c) coronal T1-weighted images. The enhancement and heterogeneity of the pituitary mass suggest hemorrhage. The optic chiasm is now visible in the coronal T1-weighted image. The pituitary stalk can also be seen deviated to the right.

A week later, after being evaluated and treated in the ER, the patient was noted to have an elevated prolactin level of 120 ng/mL (normal 4.0–15.2 ng/mL). Another MRI that was done, with contrast, revealed a pituitary mass with heterogeneous enhancement (Figure 2). There was significant reduction in size of the pituitary macroadenoma with regression from 2.5 to 1.5 cm in maximal diameter. The suprasellar extension that was present on the previous MRI had also significantly regressed. The pituitary stalk however remained deviated to the right. The patient's pituitary hormone profile was normal with the exception of an elevated prolactin likely due to stalk effect or an inefficient macroprolactinoma: TSH 4.7 mIU/L (normal 0.3–5.0 mIU/L), free thyroxine 0.9 ng/dL (normal 0.8–1.8 ng/dL), total testosterone 512 ng/dL (normal 240–950 ng/dL), bioavailable testosterone 113 ng/dL (normal 83–257 ng/dL), AM cortisol 12 mcg/dL (normal 7–25 mcg/dL), prolactin 151 ng/mL (normal 4.0–15.2 ng/mL), LH 2.8 IU/L (normal 1.8–8.6 IU/L), insulin-like growth factor 113 ng/mL (normal 75–275 ng/mL), and corticotropin 21 pg/mL (normal 10–60 pg/mL). An FSH level was not obtained.

**Figure 3:**
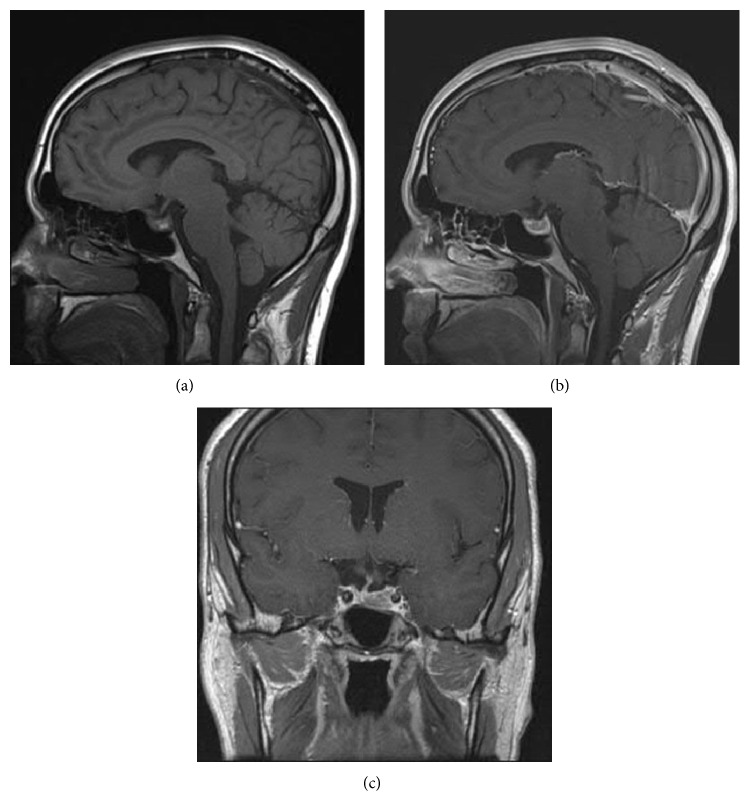
Seven weeks after the MRI in Figure 1, MRI revealed an even smaller sellar lesion on (a) sagittal precontrast T1-weighted, (b) sagittal postcontrast T1-weighted, and (c) coronal T1-weighted images. The enhancement and heterogeneity of the pituitary mass can still be seen, although less than in Figure 2. The optic chiasm is even more visible in the coronal T1-weighted image with the pitutitary mass smaller in vertical length. The pituitary stalk can still be seen deviated to the right.

With the spontaneous regression of the pituitary mass and no visual deficits on clinical exam, transsphenoidal resection of this mass was not recommended. To establish a baseline of the pituitary mass and assess any changes following the start of medical treatment, another MRI was performed. This MRI, done seven weeks after presenting to the ER, showed again further reduction in the size of the pituitary mass with it now measuring 8.1 mm in maximal diameter (Figure 3) compared to 1.5 cm on the previous MRI. There was still heterogeneous enhancement of the mass, although less than in the previous study. The pituitary stalk also still remained deviated to the right. Asymmetry of the optic chiasm was noted with the left side positioned inferiorly. This finding was believed to result from adhesions to the regressing pituitary mass. With the continued regression of the pituitary mass and the elevated prolactin levels, the decision was to proceed with cabergoline treatment.

## 3. Discussion

The start of the spontaneous regression of pituitary adenomas following apoplexy has not been clearly defined nor has the time it takes for complete resolution been clearly defined. This particular case shows that spontaneous regression can occur as early as one week following an apoplectic event. It also reveals the rate of reduction for this particular pituitary macroadenoma. At the rate it was decreasing, the mass could have potentially resolved on its own without any further treatment. This case contributes to the literature that presents spontaneous regression of pituitary adenomas.

A few of the cases showing pituitary adenomas regressing without any treatment are discussed here. Kachhara et al. present the case of a 42-year-old male with history of sudden onset of headache with associated vomiting. The patient was only treated with analgesic medications, and his symptoms resolved in one week. CT scan revealed a contrast-enhancing sellar-suprasellar mass with no extension. An MRI performed six weeks later showed complete resolution of the pituitary adenoma. The patient's pituitary macroadenoma had completely resolved with no treatment and no evidence of hypopituitarism [2]. Liu et al. present the case of a 66-year-old man with a two-month history of headaches but he experienced sudden onset of severe headache with associated vomiting and right ptosis. An MRI revealed an intrasellar mass. A pituitary hormone profile showed evidence of hypopituitarism. The patient was treated with oral prednisone and l-thyroxine with resolution of headache and eyesight improvement in two days. In three months, a repeat MRI showed complete resolution of the sellar mass [3]. Rainov and Burkert present the case of a man with a macroprolactinoma that spontaneously shrunk without any therapy after a period of six months. There is no mention of complete resolution as in the other two cases, but, like the other two cases, the pituitary mass still decreased in size [4].

In our particular case where the patient potentially has an inefficient macroprolactinoma, the mass was shown to spontaneously shrink in one week without any treatment. Although complete resolution did not occur before the decision to start cabergoline, our case provides a unique opportunity to track the spontaneous reduction in size of a pituitary mass following an apoplectic event. This case also allows us to observe how soon the reduction can occur. In one week, the patient's pituitary mass decreased from 2.5 cm (Figure 1) to 1.5 cm (Figure 2), a 40% decrease from the original size. Seven weeks after the initial event, the mass was now 8.1 mm (Figure 3) which is a 67.6% decrease from the original size. Kachhara et al. showed that a mass could completely resolve in 6 weeks while Liu et al. showed that this could occur in 3 months. Rainov and Burkert reveal an even longer period of time for shrinking to occur. Unlike these previous cases, our case provides imaging at two time points following the initial presentation of apoplectic symptoms. With only one time point, a potential time course for clot resolution cannot begin to be established. However, the two follow-up MRI studies for our patient make this possible.

Pituitary adenomas can be asymptomatic, and pituitary tumor apoplexy may be the first indication that an adenoma is present. Pituitary tumor apoplexy is thought to be due to a rapidly growing tumor that exceeds its vascular supply while another hypothesis involves the compression of portal vessels by the growing mass leading to ischemic necrosis with or without hemorrhage [5]. Apoplexy is characterized by an abrupt headache, nausea, vomiting, diminished visual acuity, visual field deficit, ocular paresis, ophthalmoplegia, and altered consciousness [5–7]. Of these symptoms, headache is the most common. In this particular case, headache was the only symptom experienced by the patient. Despite suprasellar extension of the pituitary mass touching the optic chiasm, the patient presented with no clinically apparent visual problems or deficits. If the patient had any visual abnormalities or altered mental status in association with the pituitary mass, surgical resection of the mass through a transsphenoidal approach would be indicated. This approach has been shown to improve outcomes and significantly improve visual deficits [5–7].

Surgical intervention is indicated in a majority of pituitary apoplexy cases, but conservative management can be used for select cases. Correction of electrolyte imbalances, hemodynamic stabilization, and corticosteroid repletion are among the most important initial interventions [5]. None of these were indicated in our particular case. Patients with stable, improving, or no visual symptoms can be conservatively managed. If there is biochemical evidence of a prolactinoma during the workup, medical management with a dopamine agonist has been shown to have excellent response rates [5]. This finding is why the decision was made to give cabergoline treatment to the patient in our case; however this was started at the 7-week mark. The elevation of prolactin in this patient was modest which suggests that his hyperprolactinemia may be due to stalk effect or an inefficient macroprolactinoma. Macroprolactinomas are >1.0 cm and present with symptoms of hypogonadism in approximately 80% of male patients. They are usually associated with serum prolactin levels > 250 ng/mL, and levels > 500 ng/mL make the diagnosis almost certain [8].

Stalk effect typically presents with prolactin levels of 30–200 ng/mL, and this elevation is attributed to diminished dopaminergic inhibition [9]. With findings of a deviated pituitary stalk and modest prolactin elevations, stalk effect is definitely a potential cause. However, the size of the patient's pituitary adenoma along with increasing prolactin levels can also suggest a macroprolactinoma. Hypogonadism symptoms are not present in every patient with a macroprolactinoma, and modestly elevated prolactin levels may reflect an inefficient macroprolactinoma. Definitively establishing the pituitary adenoma as a macroprolactinoma requires immunohistochemistry [10]. While we cannot determine the exact cause of our patient's hyperprolactinemia, there is no doubt that the size of his pituitary adenoma decreased.

The regression and disappearance of pituitary adenomas following apoplexy are commonly reported after patients are medically or surgically treated. However, the rapidity of resolution is unknown. Apoplexy has been shown to decrease the production of pituitary hormones released by functioning pituitary adenomas, leading to spontaneous resolution of symptoms and in some cases leading to hypopituitarism [11, 12].

The subsiding of hormonal effects associated with acromegaly and Cushing's disease has been reported [13, 14]. We did not appreciate similar findings. Our case involves a functional pituitary macroadenoma, with no symptoms associated with the elevated hormone levels, which began regressing without any treatment. Given our patient's clinical findings and endocrine profile, the appropriate management with carbergoline was initiated. If the patient had presented to us at a time point more than seven weeks after his initial presentation, the clot in his pituitary may have already fully resolved. We cannot say for sure, but, as previous cases have shown, complete resolution following an apoplectic event without significant treatment is possible. With this particular case representing the spontaneous regression of a pituitary macroadenoma associated with hyperprolactinemia, the follow-up in this patient will be critical to see how cabergoline contributes to the further regression of this tumor. Additionally, documenting the clinical course in a larger group of pituitary apoplexy patients that are monitored conservatively will prove beneficial in identifying course variation, and perhaps predict those factors that predict monitoring or surgery.
